# Supplementary material for: Resolving the full spectrum of human genome variation using Linked-Reads
Source: Genome Res. 2019 Apr;29(4):635–45. doi: 10.1101/gr.234443.118 (PMC6442396; doi:10.1101/gr.234443.118)
Supplement: Supplemental Material [file supp_gr.234443.118_Supplemental_longranger-2.2.zip › longranger-2.2.tar.bz2!/lr2.2-public/lib/stan/cmdstan/stan/doc/api/html/chi__square_8hpp_source.html]

Stan: src/stan/prob/distributions/univariate/continuous/chi\_square.hpp Source File


|  |  |
| --- | --- |
|  | Stan  2.5.0  probability, sampling & optimization |


- Main Page
- Related Pages
- Namespaces
- Classes
- Files

- File List
- File Members

All Classes Namespaces Files Functions Variables Typedefs Enumerations Enumerator Friends Macros Pages

- src
- stan
- prob
- distributions
- univariate
- continuous

chi\_square.hpp

Go to the documentation of this file.

1 #ifndef STAN\_\_PROB\_\_DISTRIBUTIONS\_\_UNIVARIATE\_\_CONTINUOUS\_\_CHI\_SQUARE\_HPP

2 #define STAN\_\_PROB\_\_DISTRIBUTIONS\_\_UNIVARIATE\_\_CONTINUOUS\_\_CHI\_SQUARE\_HPP

3

4 #include <boost/random/chi\_squared\_distribution.hpp>

5 #include <boost/random/variate\_generator.hpp>

6

7 #include <stan/agrad/partials\_vari.hpp>

8 #include <stan/math/error\_handling.hpp>

9 #include <stan/math/constants.hpp>

10 #include <stan/math/functions/multiply\_log.hpp>

11 #include <stan/math/functions/value\_of.hpp>

12 #include <stan/meta/traits.hpp>

13 #include <stan/prob/constants.hpp>

14 #include <stan/prob/traits.hpp>

15 #include <stan/prob/internal\_math.hpp>

16

17 namespace stan {

18

19  namespace prob {

20

40  template <bool propto,

41  typename T\_y, typename T\_dof>

42  typename return\_type<T\_y,T\_dof>::type

43  chi\_square\_log(const T\_y& y, const T\_dof& nu) {

44  static const char\* function = "stan::prob::chi\_square\_log(%1%)";

45

46  // check if any vectors are zero length

47  if (!(stan::length(y)

48  && stan::length(nu)))

49  return 0.0;

50

51  using stan::math::check\_positive\_finite;

52  using stan::math::check\_nonnegative;

53  using stan::math::check\_not\_nan;

54  using stan::math::check\_consistent\_sizes;

55  using stan::math::value\_of;

56

57  double logp(0.0);

58  check\_not\_nan(function, y, "Random variable", &logp);

59  check\_nonnegative(function, y, "Random variable", &logp);

60  check\_positive\_finite(function, nu, "Degrees of freedom parameter",

61  &logp);

62  check\_consistent\_sizes(function,

63  y,nu,

64  "Random variable","Degrees of freedom parameter",

65  &logp);

66

67

68  // set up template expressions wrapping scalars into vector views

69  VectorView<const T\_y> y\_vec(y);

70  VectorView<const T\_dof> nu\_vec(nu);

71  size\_t N = max\_size(y, nu);

72

73  for (size\_t n = 0; n < length(y); n++)

74  if (value\_of(y\_vec[n]) < 0)

75  return LOG\_ZERO;

76

77  // check if no variables are involved and prop-to

78  if (!include\_summand<propto,T\_y,T\_dof>::value)

79  return 0.0;

80

81  using boost::math::digamma;

82  using boost::math::lgamma;

83  using stan::math::multiply\_log;

84

85  DoubleVectorView<include\_summand<propto,T\_y,T\_dof>::value,

86  is\_vector<T\_y>::value> log\_y(length(y));

87  for (size\_t i = 0; i < length(y); i++)

88  if (include\_summand<propto,T\_y,T\_dof>::value)

89  log\_y[i] = log(value\_of(y\_vec[i]));

90

91  DoubleVectorView<include\_summand<propto,T\_y>::value,

92  is\_vector<T\_y>::value> inv\_y(length(y));

93  for (size\_t i = 0; i < length(y); i++)

94  if (include\_summand<propto,T\_y>::value)

95  inv\_y[i] = 1.0 / value\_of(y\_vec[i]);

96

97  DoubleVectorView<include\_summand<propto,T\_dof>::value,

98  is\_vector<T\_dof>::value> lgamma\_half\_nu(length(nu));

99  DoubleVectorView<!is\_constant\_struct<T\_dof>::value,

100  is\_vector<T\_dof>::value> digamma\_half\_nu\_over\_two(length(nu));

101

102  for (size\_t i = 0; i < length(nu); i++) {

103  double half\_nu = 0.5 \* value\_of(nu\_vec[i]);

104  if (include\_summand<propto,T\_dof>::value)

105  lgamma\_half\_nu[i] = lgamma(half\_nu);

106  if (!is\_constant\_struct<T\_dof>::value)

107  digamma\_half\_nu\_over\_two[i] = digamma(half\_nu) \* 0.5;

108  }

109

110

111  agrad::OperandsAndPartials<T\_y,T\_dof> operands\_and\_partials(y, nu);

112

113  for (size\_t n = 0; n < N; n++) {

114  const double y\_dbl = value\_of(y\_vec[n]);

115  const double half\_y = 0.5 \* y\_dbl;

116  const double nu\_dbl = value\_of(nu\_vec[n]);

117  const double half\_nu = 0.5 \* nu\_dbl;

118  if (include\_summand<propto,T\_dof>::value)

119  logp += nu\_dbl \* NEG\_LOG\_TWO\_OVER\_TWO - lgamma\_half\_nu[n];

120  if (include\_summand<propto,T\_y,T\_dof>::value)

121  logp += (half\_nu-1.0) \* log\_y[n];

122  if (include\_summand<propto,T\_y>::value)

123  logp -= half\_y;

124

125  if (!is\_constant\_struct<T\_y>::value) {

126  operands\_and\_partials.d\_x1[n] += (half\_nu-1.0)\*inv\_y[n] - 0.5;

127  }

128  if (!is\_constant\_struct<T\_dof>::value) {

129  operands\_and\_partials.d\_x2[n]

130  += NEG\_LOG\_TWO\_OVER\_TWO - digamma\_half\_nu\_over\_two[n] + log\_y[n]\*0.5;

131  }

132  }

133  return operands\_and\_partials.to\_var(logp);

134  }

135

136  template <typename T\_y, typename T\_dof>

137  inline

138  typename return\_type<T\_y,T\_dof>::type

139  chi\_square\_log(const T\_y& y, const T\_dof& nu) {

140  return chi\_square\_log<false>(y,nu);

141  }

142

152  template <typename T\_y, typename T\_dof>

153  typename return\_type<T\_y,T\_dof>::type

154  chi\_square\_cdf(const T\_y& y, const T\_dof& nu) {

155  static const char\* function = "stan::prob::chi\_square\_cdf(%1%)";

156

157  using stan::math::check\_positive\_finite;

158  using stan::math::check\_nonnegative;

159  using stan::math::check\_not\_nan;

160  using stan::math::check\_consistent\_sizes;

161  using stan::math::value\_of;

162

163  double cdf(1.0);

164

165  // Size checks

166  if (!(stan::length(y) && stan::length(nu)))

167  return cdf;

168

169  check\_not\_nan(function, y, "Random variable", &cdf);

170  check\_nonnegative(function, y, "Random variable", &cdf);

171  check\_positive\_finite(function, nu, "Degrees of freedom parameter", &cdf);

172  check\_consistent\_sizes(function,

173  y,nu,

174  "Random variable","Degrees of freedom parameter",

175  &cdf);

176

177  // Wrap arguments in vectors

178  VectorView<const T\_y> y\_vec(y);

179  VectorView<const T\_dof> nu\_vec(nu);

180  size\_t N = max\_size(y, nu);

181

182  agrad::OperandsAndPartials<T\_y, T\_dof>

183  operands\_and\_partials(y, nu);

184

185  // Explicit return for extreme values

186  // The gradients are technically ill-defined, but treated as zero

187  for (size\_t i = 0; i < stan::length(y); i++) {

188  if (value\_of(y\_vec[i]) == 0)

189  return operands\_and\_partials.to\_var(0.0);

190  }

191

192  // Compute CDF and its gradients

193  using boost::math::gamma\_p\_derivative;

194  using boost::math::gamma\_p;

195  using boost::math::digamma;

196  using boost::math::tgamma;

197

198  // Cache a few expensive function calls if nu is a parameter

199  DoubleVectorView<!is\_constant\_struct<T\_dof>::value,

200  is\_vector<T\_dof>::value>

201  gamma\_vec(stan::length(nu));

202  DoubleVectorView<!is\_constant\_struct<T\_dof>::value,

203  is\_vector<T\_dof>::value>

204  digamma\_vec(stan::length(nu));

205

206  if (!is\_constant\_struct<T\_dof>::value) {

207  for (size\_t i = 0; i < stan::length(nu); i++) {

208  const double alpha\_dbl = value\_of(nu\_vec[i]) \* 0.5;

209  gamma\_vec[i] = tgamma(alpha\_dbl);

210  digamma\_vec[i] = digamma(alpha\_dbl);

211  }

212  }

213

214  // Compute vectorized CDF and gradient

215  for (size\_t n = 0; n < N; n++) {

216  // Explicit results for extreme values

217  // The gradients are technically ill-defined, but treated as zero

218  if (value\_of(y\_vec[n]) == std::numeric\_limits<double>::infinity())

219  continue;

220

221  // Pull out values

222  const double y\_dbl = value\_of(y\_vec[n]);

223  const double alpha\_dbl = value\_of(nu\_vec[n]) \* 0.5;

224  const double beta\_dbl = 0.5;

225

226  // Compute

227  const double Pn = gamma\_p(alpha\_dbl, beta\_dbl \* y\_dbl);

228

229  cdf \*= Pn;

230

231  if (!is\_constant\_struct<T\_y>::value)

232  operands\_and\_partials.d\_x1[n]

233  += beta\_dbl \* gamma\_p\_derivative(alpha\_dbl, beta\_dbl \* y\_dbl)

234  / Pn;

235  if (!is\_constant\_struct<T\_dof>::value)

236  operands\_and\_partials.d\_x2[n]

237  -= 0.5 \* stan::math::gradRegIncGamma(alpha\_dbl, beta\_dbl

238  \* y\_dbl, gamma\_vec[n],

239  digamma\_vec[n]) / Pn;

240  }

241

242  if (!is\_constant\_struct<T\_y>::value)

243  for (size\_t n = 0; n < stan::length(y); ++n)

244  operands\_and\_partials.d\_x1[n] \*= cdf;

245  if (!is\_constant\_struct<T\_dof>::value)

246  for (size\_t n = 0; n < stan::length(nu); ++n)

247  operands\_and\_partials.d\_x2[n] \*= cdf;

248

249  return operands\_and\_partials.to\_var(cdf);

250  }

251

252  template <typename T\_y, typename T\_dof>

253  typename return\_type<T\_y,T\_dof>::type

254  chi\_square\_cdf\_log(const T\_y& y, const T\_dof& nu) {

255  static const char\* function = "stan::prob::chi\_square\_cdf\_log(%1%)";

256

257  using stan::math::check\_positive\_finite;

258  using stan::math::check\_nonnegative;

259  using stan::math::check\_not\_nan;

260  using stan::math::check\_consistent\_sizes;

261  using stan::math::value\_of;

262

263  double cdf\_log(0.0);

264

265  // Size checks

266  if (!(stan::length(y) && stan::length(nu)))

267  return cdf\_log;

268

269  check\_not\_nan(function, y, "Random variable", &cdf\_log);

270  check\_nonnegative(function, y, "Random variable", &cdf\_log);

271  check\_positive\_finite(function, nu, "Degrees of freedom parameter",

272  &cdf\_log);

273  check\_consistent\_sizes(function,

274  y,nu,

275  "Random variable","Degrees of freedom parameter",

276  &cdf\_log);

277

278  // Wrap arguments in vectors

279  VectorView<const T\_y> y\_vec(y);

280  VectorView<const T\_dof> nu\_vec(nu);

281  size\_t N = max\_size(y, nu);

282

283  agrad::OperandsAndPartials<T\_y, T\_dof>

284  operands\_and\_partials(y, nu);

285

286  // Explicit return for extreme values

287  // The gradients are technically ill-defined, but treated as zero

288  for (size\_t i = 0; i < stan::length(y); i++) {

289  if (value\_of(y\_vec[i]) == 0)

290  return operands\_and\_partials.to\_var(stan::math::negative\_infinity());

291  }

292

293  // Compute cdf\_log and its gradients

294  using boost::math::gamma\_p\_derivative;

295  using boost::math::gamma\_p;

296  using boost::math::digamma;

297  using boost::math::tgamma;

298

299  // Cache a few expensive function calls if nu is a parameter

300  DoubleVectorView<!is\_constant\_struct<T\_dof>::value,

301  is\_vector<T\_dof>::value>

302  gamma\_vec(stan::length(nu));

303  DoubleVectorView<!is\_constant\_struct<T\_dof>::value,

304  is\_vector<T\_dof>::value>

305  digamma\_vec(stan::length(nu));

306

307  if (!is\_constant\_struct<T\_dof>::value) {

308  for (size\_t i = 0; i < stan::length(nu); i++) {

309  const double alpha\_dbl = value\_of(nu\_vec[i]) \* 0.5;

310  gamma\_vec[i] = tgamma(alpha\_dbl);

311  digamma\_vec[i] = digamma(alpha\_dbl);

312  }

313  }

314

315  // Compute vectorized cdf\_log and gradient

316  for (size\_t n = 0; n < N; n++) {

317  // Explicit results for extreme values

318  // The gradients are technically ill-defined, but treated as zero

319  if (value\_of(y\_vec[n]) == std::numeric\_limits<double>::infinity())

320  return operands\_and\_partials.to\_var(0.0);

321

322  // Pull out values

323  const double y\_dbl = value\_of(y\_vec[n]);

324  const double alpha\_dbl = value\_of(nu\_vec[n]) \* 0.5;

325  const double beta\_dbl = 0.5;

326

327  // Compute

328  const double Pn = gamma\_p(alpha\_dbl, beta\_dbl \* y\_dbl);

329

330  cdf\_log += log(Pn);

331

332  if (!is\_constant\_struct<T\_y>::value)

333  operands\_and\_partials.d\_x1[n]

334  += beta\_dbl \* gamma\_p\_derivative(alpha\_dbl, beta\_dbl \* y\_dbl)

335  / Pn;

336  if (!is\_constant\_struct<T\_dof>::value)

337  operands\_and\_partials.d\_x2[n]

338  -= 0.5 \* stan::math::gradRegIncGamma(alpha\_dbl, beta\_dbl

339  \* y\_dbl, gamma\_vec[n],

340  digamma\_vec[n]) / Pn;

341  }

342

343  return operands\_and\_partials.to\_var(cdf\_log);

344  }

345

346  template <typename T\_y, typename T\_dof>

347  typename return\_type<T\_y,T\_dof>::type

348  chi\_square\_ccdf\_log(const T\_y& y, const T\_dof& nu) {

349  static const char\* function = "stan::prob::chi\_square\_ccdf\_log(%1%)";

350

351  using stan::math::check\_positive\_finite;

352  using stan::math::check\_nonnegative;

353  using stan::math::check\_not\_nan;

354  using stan::math::check\_consistent\_sizes;

355  using stan::math::value\_of;

356

357  double ccdf\_log(0.0);

358

359  // Size checks

360  if (!(stan::length(y) && stan::length(nu)))

361  return ccdf\_log;

362

363  check\_not\_nan(function, y, "Random variable", &ccdf\_log);

364  check\_nonnegative(function, y, "Random variable", &ccdf\_log);

365  check\_positive\_finite(function, nu, "Degrees of freedom parameter",

366  &ccdf\_log);

367  check\_consistent\_sizes(function,

368  y,nu,

369  "Random variable","Degrees of freedom parameter",

370  &ccdf\_log);

371

372  // Wrap arguments in vectors

373  VectorView<const T\_y> y\_vec(y);

374  VectorView<const T\_dof> nu\_vec(nu);

375  size\_t N = max\_size(y, nu);

376

377  agrad::OperandsAndPartials<T\_y, T\_dof>

378  operands\_and\_partials(y, nu);

379

380  // Explicit return for extreme values

381  // The gradients are technically ill-defined, but treated as zero

382  for (size\_t i = 0; i < stan::length(y); i++) {

383  if (value\_of(y\_vec[i]) == 0)

384  return operands\_and\_partials.to\_var(0.0);

385  }

386

387  // Compute ccdf\_log and its gradients

388  using boost::math::gamma\_p\_derivative;

389  using boost::math::gamma\_p;

390  using boost::math::digamma;

391  using boost::math::tgamma;

392

393  // Cache a few expensive function calls if nu is a parameter

394  DoubleVectorView<!is\_constant\_struct<T\_dof>::value,

395  is\_vector<T\_dof>::value>

396  gamma\_vec(stan::length(nu));

397  DoubleVectorView<!is\_constant\_struct<T\_dof>::value,

398  is\_vector<T\_dof>::value>

399  digamma\_vec(stan::length(nu));

400

401  if (!is\_constant\_struct<T\_dof>::value) {

402  for (size\_t i = 0; i < stan::length(nu); i++) {

403  const double alpha\_dbl = value\_of(nu\_vec[i]) \* 0.5;

404  gamma\_vec[i] = tgamma(alpha\_dbl);

405  digamma\_vec[i] = digamma(alpha\_dbl);

406  }

407  }

408

409  // Compute vectorized ccdf\_log and gradient

410  for (size\_t n = 0; n < N; n++) {

411  // Explicit results for extreme values

412  // The gradients are technically ill-defined, but treated as zero

413  if (value\_of(y\_vec[n]) == std::numeric\_limits<double>::infinity())

414  return operands\_and\_partials.to\_var(stan::math::negative\_infinity());

415

416  // Pull out values

417  const double y\_dbl = value\_of(y\_vec[n]);

418  const double alpha\_dbl = value\_of(nu\_vec[n]) \* 0.5;

419  const double beta\_dbl = 0.5;

420

421  // Compute

422  const double Pn = 1.0 - gamma\_p(alpha\_dbl, beta\_dbl \* y\_dbl);

423

424  ccdf\_log += log(Pn);

425

426  if (!is\_constant\_struct<T\_y>::value)

427  operands\_and\_partials.d\_x1[n]

428  -= beta\_dbl \* gamma\_p\_derivative(alpha\_dbl, beta\_dbl \* y\_dbl)

429  / Pn;

430  if (!is\_constant\_struct<T\_dof>::value)

431  operands\_and\_partials.d\_x2[n]

432  += 0.5 \* stan::math::gradRegIncGamma(alpha\_dbl, beta\_dbl

433  \* y\_dbl, gamma\_vec[n],

434  digamma\_vec[n]) / Pn;

435  }

436

437  return operands\_and\_partials.to\_var(ccdf\_log);

438  }

439

440  template <class RNG>

441  inline double

442  chi\_square\_rng(const double nu,

443  RNG& rng) {

444  using boost::variate\_generator;

445  using boost::random::chi\_squared\_distribution;

446

447  static const char\* function = "stan::prob::chi\_square\_rng(%1%)";

448

449  using stan::math::check\_positive\_finite;

450

451  check\_positive\_finite(function, nu, "Degrees of freedom parameter",

452  (double\*)0);

453

454

455  variate\_generator<RNG&, chi\_squared\_distribution<> >

456  chi\_square\_rng(rng, chi\_squared\_distribution<>(nu));

457  return chi\_square\_rng();

458  }

459  }

460 }

461

462 #endif

463

multiply\_log.hpp

traits.hpp

stan::agrad::tgamma

fvar< T > tgamma(const fvar< T > &x)

**Definition:** tgamma.hpp:15

stan::agrad::OperandsAndPartials::to\_var

T\_return\_type to\_var(double logp)

**Definition:** partials\_vari.hpp:138

stan::math::check\_positive\_finite

bool check\_positive\_finite(const char \*function, const T\_y &y, const char \*name, T\_result \*result)

**Definition:** check\_positive\_finite.hpp:12

error\_handling.hpp

stan::math::multiply\_log

boost::math::tools::promote\_args< T\_a, T\_b >::type multiply\_log(const T\_a a, const T\_b b)

Calculated the value of the first argument times log of the second argument while behaving properly w...

**Definition:** multiply\_log.hpp:51

stan::length

size\_t length(const T &)

**Definition:** traits.hpp:159

stan::DoubleVectorView

DoubleVectorView allocates double values to be used as intermediate values.

**Definition:** traits.hpp:358

partials\_vari.hpp

stan::agrad::value\_of

T value\_of(const fvar< T > &v)

Return the value of the specified variable.

**Definition:** value\_of.hpp:16

constants.hpp

stan::prob::chi\_square\_log

return\_type< T\_y, T\_dof >::type chi\_square\_log(const T\_y &y, const T\_dof &nu)

The log of a chi-squared density for y with the specified degrees of freedom parameter.

**Definition:** chi\_square.hpp:43

stan::is\_vector

**Definition:** traits.hpp:83

stan::agrad::lgamma

fvar< T > lgamma(const fvar< T > &x)

**Definition:** lgamma.hpp:15

stan::agrad::OperandsAndPartials

A variable implementation that stores operands and derivatives with respect to the variable...

**Definition:** partials\_vari.hpp:76

traits.hpp

stan::return\_type::type

boost::math::tools::promote\_args< typename scalar\_type< T1 >::type, typename scalar\_type< T2 >::type, typename scalar\_type< T3 >::type, typename scalar\_type< T4 >::type, typename scalar\_type< T5 >::type, typename scalar\_type< T6 >::type >::type type

**Definition:** traits.hpp:406

stan::is\_constant\_struct

Metaprogram to determine if a type has a base scalar type that can be assigned to type double...

**Definition:** traits.hpp:57

stan::math::value\_of

double value\_of(const T x)

Return the value of the specified scalar argument converted to a double value.

**Definition:** value\_of.hpp:24

stan::prob::include\_summand

Template metaprogram to calculate whether a summand needs to be included in a proportional (log) prob...

**Definition:** traits.hpp:35

stan::agrad::OperandsAndPartials::d\_x2

VectorView< double \*, is\_vector< T2 >::value, is\_constant\_struct< T2 >::value > d\_x2

**Definition:** partials\_vari.hpp:83

stan::prob::chi\_square\_cdf\_log

return\_type< T\_y, T\_dof >::type chi\_square\_cdf\_log(const T\_y &y, const T\_dof &nu)

**Definition:** chi\_square.hpp:254

stan::math::gradRegIncGamma

double gradRegIncGamma(double a, double z, double g, double dig, double precision=1e-6)

**Definition:** internal\_math.hpp:181

stan::math::check\_nonnegative

bool check\_nonnegative(const char \*function, const T\_y &y, const char \*name, T\_result \*result)

**Definition:** check\_nonnegative.hpp:52

stan::prob::chi\_square\_ccdf\_log

return\_type< T\_y, T\_dof >::type chi\_square\_ccdf\_log(const T\_y &y, const T\_dof &nu)

**Definition:** chi\_square.hpp:348

stan::math::check\_consistent\_sizes

bool check\_consistent\_sizes(const char \*function, const T1 &x1, const T2 &x2, const char \*name1, const char \*name2, T\_result \*result)

**Definition:** check\_consistent\_sizes.hpp:13

stan::max\_size

size\_t max\_size(const T1 &x1, const T2 &x2)

**Definition:** traits.hpp:191

stan::agrad::gamma\_p

fvar< T > gamma\_p(const fvar< T > &x1, const fvar< T > &x2)

**Definition:** gamma\_p.hpp:16

internal\_math.hpp

stan::math::check\_not\_nan

bool check\_not\_nan(const char \*function, const T\_y &y, const char \*name, T\_result \*result)

Checks if the variable y is nan.

**Definition:** check\_not\_nan.hpp:56

stan::agrad::digamma

fvar< T > digamma(const fvar< T > &x)

**Definition:** digamma.hpp:16

value\_of.hpp

stan::agrad::OperandsAndPartials::d\_x1

VectorView< double \*, is\_vector< T1 >::value, is\_constant\_struct< T1 >::value > d\_x1

**Definition:** partials\_vari.hpp:82

constants.hpp

stan::agrad::log

fvar< T > log(const fvar< T > &x)

**Definition:** log.hpp:15

stan::VectorView

VectorView is a template metaprogram that takes its argument and allows it to be used like a vector...

**Definition:** traits.hpp:275

stan::prob::chi\_square\_cdf

return\_type< T\_y, T\_dof >::type chi\_square\_cdf(const T\_y &y, const T\_dof &nu)

Calculates the chi square cumulative distribution function for the given variate and degrees of freed...

**Definition:** chi\_square.hpp:154

stan::math::negative\_infinity

double negative\_infinity()

Return negative infinity.

**Definition:** constants.hpp:123

stan::prob::chi\_square\_rng

double chi\_square\_rng(const double nu, RNG &rng)

**Definition:** chi\_square.hpp:442


---

[ Stan Home Page ]

*© 2011–2014,
Stan Development Team.*
